# Applying the estimand and target trial frameworks to external control analyses using observational data: a case study in the solid tumor setting

**DOI:** 10.3389/fphar.2024.1223858

**Published:** 2024-01-26

**Authors:** Letizia Polito, Qixing Liang, Navdeep Pal, Philani Mpofu, Ahmed Sawas, Olivier Humblet, Kaspar Rufibach, Dominik Heinzmann

**Affiliations:** ^1^ Product Development Data Sciences, F Hoffmann-La Roche Ltd., Basel, Switzerland; ^2^ Flatiron Health, Inc., New York, NY, United States; ^3^ Genentech, Inc., San Francisco, CA, United States

**Keywords:** causal inference, estimand framework, target trial emulation framework, external control, oncology, real-world data

## Abstract

**Introduction:** In causal inference, the correct formulation of the scientific question of interest is a crucial step. The purpose of this study was to apply causal inference principles to external control analysis using observational data and illustrate the process to define the estimand attributes.

**Methods:** This study compared long-term survival outcomes of a pooled set of three previously reported randomized phase 3 trials studying patients with metastatic non-small cell lung cancer receiving front-line chemotherapy and similar patients treated with front-line chemotherapy as part of routine clinical care. Causal inference frameworks were applied to define the estimand aligned with the research question and select the estimator to estimate the estimand of interest.

**Results:** The estimand attributes of the ideal trial were defined using the estimand framework. The target trial framework was used to address specific issues in defining the estimand attributes using observational data from a nationwide electronic health record-derived de-identified database. The two frameworks combined allow to clearly define the estimand and the aligned estimator while accounting for key baseline confounders, index date, and receipt of subsequent therapies. The hazard ratio estimate (point estimate with 95% confidence interval) comparing the randomized clinical trial pooled control arm with the external control was close to 1, which is indicative of similar survival between the two arms.

**Discussion:** The proposed combined framework provides clarity on the causal contrast of interest and the estimator to adopt, and thus facilitates design and interpretation of the analyses.

## 1 Introduction

Several causal inference frameworks, including the estimand framework (EF), target trial emulation framework (TTF), and PICO framework, exist to help define a precise scientific question for comparative assessments in clinical research and development (Goetghebeur et al., 2020). There are overlapping but complementary elements in these frameworks, suggesting the potential for a combined application; however, this presents challenges to investigators as there are limited practical examples and guidance for the combined application of the frameworks.

The EF has increasingly been adopted by health authorities and pharmaceutical companies since its initial publication in August 2017 (Food and Drug Administration, 2021). The EF enables researchers to specify a precise scientific question by using five attributes that define the estimand (i.e., the treatment effect of interest or the “what to estimate”). These five interrelated attributes are population, treatment, variable of interest (endpoint), intercurrent event handling, and the summary measure. An intercurrent event is an event occurring after treatment initiation that affects either the interpretation or the existence of the measurements associated with the endpoint. For example, if performing a comparative assessment on overall survival (OS) between two different treatments, candidates for intercurrent events include, among others, early discontinuation of treatment or treatment switching after disease progression. In general, the definition of the estimand comes first and is derived from the scientific objective of the trial or study. Together with considerations about missing data, the framework then informs the choice of the estimator. The addendum acknowledges that usually an iterative process will be necessary to reach an estimand that is clinically relevant for decision making and for which a reliable estimate can be computed. If it is not possible to develop an appropriate trial design or to derive an adequately reliable estimate for a particular estimand, an alternative estimand, trial design, or method of analysis may need to be considered. However, practical examples in the literature describing such an iterative process to redefine an initial target estimand, while also considering aspects of identifiability (and hence the estimator) are limited. While the focus of the ICH E9 addendum is on randomized clinical trials (RCTs), the principles are also applicable whenever estimating a treatment effect (i.e., non-randomized studies). However, estimation of a causal effect from observational data, compared to RCT data, often has additional challenges. Namely, observational data is more often incomplete, heterogeneous, and subject to different types of measurement errors and biases (e.g., selection bias, bias due to baseline confounding, and the ability to correctly define the index date for comparison) (Liu and Panagiotakos, 2023).

The TTF is another causal framework that can be used to specify the scientific question more precisely in a comparative assessment (Hernán and Robins, 2016). TTF complements the EF by addressing gaps related to the analysis of observational data and applies design principles of an RCT to the specific setting of a non-randomized comparative assessment (Hernán et al., 2008; Cain et al., 2016; Hernán and Robins, 2016; Petito et al., 2020). TTF entails defining a hypothetical randomized trial to address a precise scientific question and then further specifying how it can be emulated (i.e., approximated) by non-randomized data. The essential components of a target trial protocol are eligibility criteria, treatment strategies, treatment assignment, start/end of follow-up, outcomes, causal contrasts, and the analysis approach (estimator) (Hernán and Robins, 2016). The framework can also be utilized when a combination of clinical trial and observational data are used, for example, to contextualize a single-arm clinical trial with observational data (Thomas et al., 2021). Combining the EF and the TTF provides a structured approach to enhance the scientific rigor for causal inference for observational and/or non-randomized data. Together they bring more transparency to the causal estimand, which supports specifying the attributes of the estimand and the assumptions made to draw causal conclusions.

Another framework that aims to define the precise scientific question includes the PICO framework (Schardt et al., 2007), traditionally used in epidemiology for observational studies. The EF and TTF extend the PICO framework, with the former adding intercurrent events and ensuring that the population-level summary measure is made explicit, and the latter adding the causal contrast, assignment procedures, and the start/end of follow-up. By explicitly calling out these key elements, the treatment effect can be adequately defined.

An important goal in pharmacoepidemiology is to assess whether observational data (including electronic health record [EHR]-derived data) can emulate (and thus supplement or replace, e.g., for regulatory decision-making) the control arm of a RCT, while acknowledging that there are differences between clinical trial and routine clinical settings, at baseline and post-baseline, that may have an impact on the outcome independently from the treatment received. In this study we jointly apply the EF and TTF to perform a comparative effectiveness assessment in patients with non-small cell lung cancer (NSCLC) using data from a set of pooled control arms of three RCTs as well as EHR-derived de-identified observational data (West et al., 2019; Jotte et al., 2020; Nishio et al., 2021). The objective of our case study was to determine whether there is a difference in OS between patients with metastatic NSCLC receiving front-line chemotherapy in pivotal trials *versus* patients with metastatic NSCLC who received front-line chemotherapy as part of routine care, had patients not received a subsequent therapy. This case study aims to illustrate the application of the EF to observational data, and the benefits of complementing the EF with the TTF to account for specific challenges in observational data that are not directly addressed by the EF (and *vice versa*, as the handling of intercurrent events is not explicitly addressed in the TTF). The iterative process (as indicated in the EF) to arrive at the final scientific question is illustrated in the Methods section. In sum, the present study provides insights into where the two frameworks are complementary and provides a practical example of their joint application.

## 2 Materials and methods

### 2.1 Applying the frameworks to the research question

Before discussing details of the joint application of the EF and TTF to define the final scientific question, we want to provide insights and stepwise practical guidance on the iterative process outlined in the EF to arrive at the final question:

Step 1: determine the comparison of interest.

Step 2: develop the scientific question.

Step 3: discuss the implications of estimating the estimand aligned with the scientific question, thinking in terms of estimand attributes, including potential intercurrent events and the consequences of different strategies used to handle them.

Step 4: refine the scientific question if needed and iterate Steps 3–4 until the question is clear enough to leave no ambiguity about the estimand.

Applying these steps, we were interested in comparing the treatment effect of the same front-line treatment given in a clinical trial *versus* in the clinical practice when subsequent treatments would be similar. We started with the scientific question: “Is there a difference in OS between patients with metastatic NSCLC receiving front-line chemotherapy in pivotal trials *versus* patients with metastatic NSCLC who received front-line chemotherapy as part of routine care?” EHR-derived observational data from routine clinical practice suggests a larger heterogeneity in subsequent second-line cancer treatments as compared to the clinical trial setting (Signorovitch et al., 2022). This difference in the range of potential subsequent therapies may introduce complexities in estimating causal treatment effects for longer-term outcomes such as OS and ultimately complicate interpretations. Therefore, the initial research question has been iterated to: “Is there a difference in OS between patients with metastatic NSCLC receiving front-line chemotherapy in pivotal trials *versus* patients with metastatic NSCLC who received front-line chemotherapy as part of routine care, had patients not received a subsequent therapy?” Hence, instead of considering the entire treatment strategy (front-line and subsequent therapy) which is complicated by heterogeneity in subsequent therapies among clinical trial and clinical practice settings, the iteration resulted in the scientific question of treatment effect of the front-line regimens.

Now we focus on jointly applying the EF and TTF to the final scientific question. Table 1 displays the EF/TTF attributes that define the estimand aligned with the scientific research question. We define the hypothetical target trial structured according to the EF and the study that attempts to emulate it, leveraging elements from the EF and TTF. The average treatment effect on the treated (ATT) is the estimand of primary interest. This is the treatment effect difference of using front-line chemotherapy in a clinical trial *versus* in clinical practice, where the target population is defined by the population of the three clinical trials.

**TABLE 1:** EF/TTF attributes based on the scientific research question.

Scientific research question			
Would there be a difference in OS between patients with metastatic NSCLC receiving front-line chemotherapy (control arms) in IMpower trials (130, 131 and 132) vs. patients with metastatic NSCLC who received front-line chemotherapy as part of routine care, had patients not received a subsequent therapy?			
*EF/TTF Attributes*	Target trial	Emulation of the target trial	Assumptions
**Target population/Eligibility criteria**	Metastatic squamous and non-squamous NSCLC patients, 18 years of age or older, with ECOG PS 0,1 and with adequate hematological and end-organ function. The population is defined through the common I/E criteria of IMpower130, 131 and 132 (limited to those criteria applicable retrospectively to observational data). To align the I/E criteria of the 3 trials, and to reflect the targeted population treated with 1L chemotherapy, patients with a sensitizing mutation in the *EGFR* gene or an *ALK* fusion oncogene were excluded	Same as the target trial for the RCT arm, with some assumptions for the OC arm	Observational data does not perfectly emulate the trial I/E criteria. We attempt to define the study cohort that best approximates the target population by including additional rules
			• Time window for the eligibility assessment (ECOG PS, lab values, biomarker)
			• How to handle missing values (ECOG PS, lab values, biomarker)
			○ Excluding patients with missing value may introduce selection bias
			• Rules to account for difference between trial structured visits and routine clinical care
			○ E.g., Patients with structured activity within 90 days of advanced diagnosis
**Treatment/Treatment strategies**	The investigational arm (pooled trial control arms) and the OC arm received the following chemotherapies	Same as the target trial with some assumptions for both arms	Assumption on treatment: • For this study we assume equivalence of nab-paclitaxel and paclitaxel. However, the two molecules are known to have different safety profiles. The decision to include paclitaxel was to limit treatment assignment bias since nab-paclitaxel is not the standard of care in the real world while it was adopted in IMpower trials
	Patients with non-squamous NSCLC		
	-Pemetrexed + cisplatin/carboplatin		
	-nab-paclitaxel/paclitaxel + carboplatin*		
	Patients with squamous NSCLC		
	-nab-paclitaxel/paclitaxel + carboplatin*		
	The investigational arm received care according to the trial protocol, whereas the comparator arm received care according to routine clinical practice		
**Endpoint/Outcomes**	OS	Same as the target trial	None. The validity of the rwOS from Flatiron Health has been demonstrated (Zhang et al., 2021) against clinical trial OS as the gold standard to capture death occurrence. For this reason, in this study we refer to OS and not to rwOS for routine clinical practice
**Intercurrent events (IE) and strategy/Causal contrast**	IE: Receipt of any subsequent cancer therapy	Same as the target trial	None
	Strategy: hypothetical		
	Causal contrast: Per-protocol effect of adhering to treatment after initiation. Receipt of any subsequent cancer therapy is a deviation from the study protocol.		
**Population-level summary/analysis plan**	HR with 95% CI	Same as the target trial	None
**Assignment procedures**	Participants were randomly assigned to one of the two treatment settings	Randomization is emulated by weighting observations for the inverse probability of treatment setting assignment following some assumptions	Clinical assumptions
			Treatment setting assignment was assumed to be conditional on the following baseline covariates
			• Age, gender, race, metastatic tumor type (*de novo* Stage IV/recurrent disease), time from initial diagnosis to index date, smoking history, histology, and treatment type
			Statistical assumptions
			Statistical assumptions include consistency, conditional exchangeability, positivity and correct model specification. These are explained in the text
**Start/end follow-up**	Start of follow-up occurs at the time when the treatment is assigned (i.e., when eligibility is met) End of follow-up is reported in Supplementary Table 1	Same as target trial. To emulate the start of follow up for the OC arm, some assumptions are needed. To emulate the end of follow up we truncated the follow-up time at Month 21 because there were few patients remaining in the RCT arm after Month 21	For the OC arm, the actual start of follow-up occurs at the time when the treatment is initiated (dose 1 cycle 1)
			The risk of comparing different time zero is to introduce immortal time bias. This cannot be quantified. The primary estimate is unbiased if the following assumptions are met.
			Assumptions in the OC
			• There are no reasons for a patient to not initiate treatment other than death once assigned to treatment
			• Death is unlikely to have occurred in between assignment and start of treatment because we assume
			○ The time between assignment and start of therapy is short
			○ mNSCLC is a disease with no rapid course in first line
			No assumption for RCT. We verified that
			• All patients assigned to treatment started treatment
			• Median time between assignment and start of therapy was 2 days

Notes: 1L, first-line therapy; ALK, anaplastic lymphoma kinase; CI, confidence interval; EGFR, epidermal growth factor receptor; HR, hazard ratio; I/E, inclusion and exclusion; mNSCLC, metastatic non-small cell lung cancer; NSCLC, non-small cell lung cancer; OC, observational comparator; OS, overall survival; RCT, randomized clinical trials; rwOS, real-world overall survival.

### 2.2 Data source

#### 2.2.1 Clinical trial data

Individual patient-level data (IPD) were used from Roche-sponsored phase III, open-label randomized clinical trials IMpower130 (ClinicalTrials.gov identifier: NCT02367781), 131 (ClinicalTrials.gov identifier: NCT02367794), and 132 (ClinicalTrials.gov identifier: NCT02657434). Methods and primary findings have been previously reported (West et al., 2019; Jotte et al., 2020; Nishio et al., 2021). These three trials included patients who were chemotherapy-naive and had stage IV NSCLC. OS was the primary endpoint for the three trials. To address the objective of the present study, only the IPD from the control arms were used. The control arms received platinum-based chemotherapy as follows:

• IMpower130 included patients with non-squamous NSCLC treated with carboplatin plus nab-paclitaxel
• IMpower131 included patients with squamous NSCLC treated with carboplatin plus nab-paclitaxel
• IMpower132 included patients with non-squamous NSCLC treated with carboplatin or cisplatin plus pemetrexed

As these three clinical trial control arms had similar settings in terms of disease, therapy, and inclusion/exclusion criteria and had similar survival outcomes such as median survival time (Supplementary Figure S1), they were pooled together to increase the sample size and are collectively referred to as the RCT arm in this study.

#### 2.2.2 Observational data

The observational comparator (OC) arm of this study was developed using the nationwide (US-based) Flatiron Health EHR-derived de-identified database. This longitudinal database is comprised of patient-level structured (e.g., laboratory values and prescribed treatments) and unstructured data (e.g., biomarker reports) curated from technology-enabled chart abstraction from physicians’ notes and other documents (Birnbaum et al., 2020; Ma et al., 2020). During the study period, the de-identified data originated from approximately 280 cancer clinics (approximately 800 sites of care, primarily community-based cancer centers). The studies involving human participants were reviewed and approved by the IRB of WCG IRB and included a waiver of informed consent.

### 2.3 Cohort selection/study sample

The OC cohort was selected to align, as closely as possible, to the eligibility (inclusion/exclusion) criteria of the three clinical trials, which reflected the eligibility criteria of the target trial (Table 1 and Supplementary Table S2). This deliberate selection allowed us to define a pooled sample of one common target population. To be eligible for entry into the de-identified database, the patient’s EHR must include >1 visit to a community oncology clinic and have confirmation of an advanced NSCLC diagnosis and histological subtype (squamous vs. non-squamous histology) through a review of unstructured data (i.e., clinical notes, radiology reports, or pathology reports). A front-line therapy start date for advanced or metastatic NSCLC on or after 16 April 2015, and on or before 31 May 2017, to match the clinical trials’ start and end dates of enrollment was also required. Patients with an Eastern Cooperative Oncology Group performance status (ECOG PS) of 0, 1, or unknown were included. Patients had to have received at least one administration of the regimens of interest (i.e., carboplatin plus paclitaxel/nab-paclitaxel, carboplatin, or cisplatin plus pemetrexed). Patients who had potentially incomplete historical treatment data (i.e., >90-day gap between advanced diagnosis and structured activity in the EHR), therapy within 6 months before the start of front-line therapy for advanced-stage disease, receipt of a clinical study drug, or multiple primary tumors were excluded. Patients with missing information or known to have a sensitizing mutation in the epidermal growth factor receptor (*EGFR*) gene or anaplastic lymphoma kinase (*ALK*) fusion oncogene were excluded. All patients were followed until 18 July 2019. Detailed inclusion/exclusion criteria were included in Supplementary Table S2.

### 2.4 Statistical analyses

**FIGURE 1:**
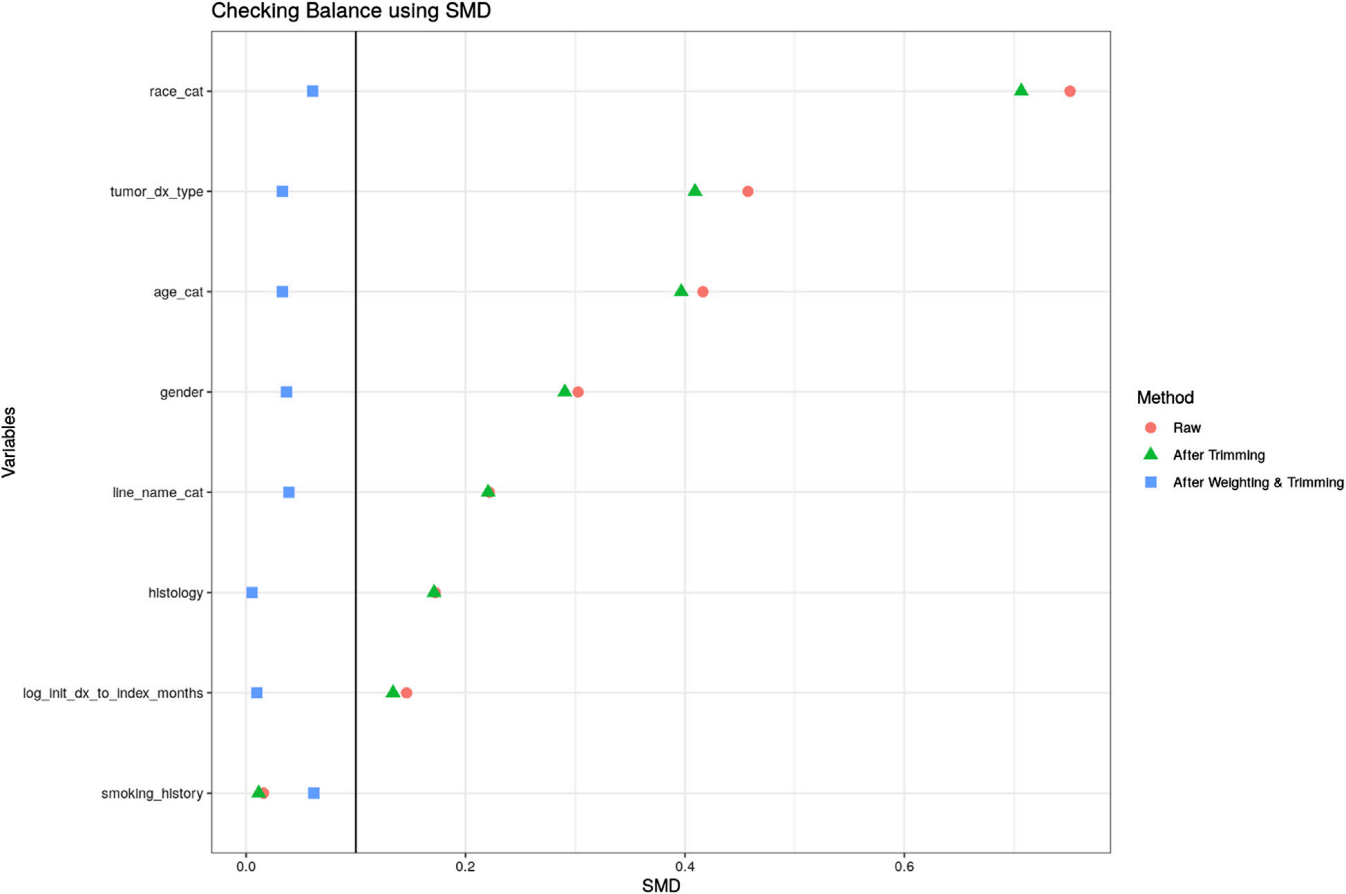
Covariate balance after IPTW-ATT.

We applied the following estimation approach to target the ATT estimand with attributes as specified in Table 1. First, the inverse probability of treatment weighting (IPTW) method was used to balance baseline patient characteristics between the RCT arm and the OC arm. A multiple logistic regression model was used to estimate propensity scores (PS) that are defined as probabilities of being assigned to the RCT arm conditional on all confounders that were selected based on clinical experts’ knowledge and availability of the relevant variables. Because we target the ATT as described above, patients from clinical trials were given a weight of one. In contrast, patients’ weights from the OC cohort were defined as the ratio of the estimated PS to one minus the estimated PS (i.e., odds of being treated in the clinical setting). We refer to these weights as IPTW-ATT weights. Before and after IPTW-ATT weighting, differences in baseline characteristics were assessed through standardized mean and proportion differences (SMD) (Table 2; Figure 1). Patient characteristics were considered statistically different if SMD ≥0.10 (Austin and Stuart, 2015). In addition, we examined the propensity score distribution to ensure a reasonable overlap between the two cohorts. The weighted population was used in the subsequent analyses.

**TABLE 2:** Baseline characteristics.

Variable	Categories	RCT arm, N = 849	OC arm, N = 3340	SMD
Age group (years), n (%)	<65	435 (51.2)	1222 (36.6)	0.42
	≥65 and <75	322 (37.9)	1268 (38.0)	
	≥75	92 (10.8)	850 (25.4)	
Gender, n (%)	Female	248 (29.2)	1457 (43.6)	0.30
Race, n (%)	Asian	105 (12.4)	46 (1.4)	0.75
	White	699 (82.3)	2373 (71.0)	
	Other	45 (5.3)	921 (27.6)	
ECOG PS, n (%)	0	314 (37.0)	714 (21.4)	0.05a
	1	532 (62.7)	1179 (35.3)	
	Unknown	2 (0.2)	1447 (43.3)	
Tumor diagnosis type, n (%)	*De novo* Stage IV	706 (83.2)	2118 (63.4)	0.46
	Recurrent disease	143 (16.8)	1221 (36.6)	
Smoking history, n (%)	No	69 (8.1)	257 (7.7)	0.02
	Yes	780 (91.9)	3070 (91.9)	
	Unknown	0 (0.0)	13 (0.4)	
Histology, n (%)	Non-squamous	509 (60.0)	2278 (68.2)	0.17
	Squamous	340 (40.0)	1062 (31.8)	
Time from initial diagnosis to index date (months), median [IQR]		1.41 [0.92, 2.89]	1.25 [0.79, 2.27]	0.15
Treatment, n (%)	Carboplatin + Pacli/Nab-pacli	568 (66.9)	1877 (56.2)	0.22
	Platinum + Pemetrexed	281 (33.1)	1463 (43.8)	

Notes: ECOG PS, eastern cooperative group performance status; OC, observational comparator; RCT, randomized clinical trial; SMD, standardized mean and proportion differences.

^a^ The “unknown” category was not considered for SMD, calculation.

^b^ ECOG PS, variable was not included in the propensity score model because of the high proportion of missing values.

Secondly, the inverse probability of censoring weighting (IPCW) method was used to handle informative censoring introduced by censoring patients upon the occurrence of the intercurrent event of interest, i.e., receipt of any subsequent cancer therapy, as per the hypothetical strategy of handling intercurrent events (Table 1). We artificially censored patients at the time of receipt of first second-line treatment and used the IPCW method to estimate weights for the follow-up information for the remaining patients using both baseline and time-varying variables, which are likely to impact treatment switching based on clinical experts’ knowledge to adjust for any potential confounding created by the artificial censoring. Specifically, we fit a Cox model for each arm that was used to estimate the probability of not being censored by time (t) given baseline and time-varying covariates (listed in Table 2) for the specific group. The IPCW weights are calculated as the inverse of the conditional probability of not being censored. We truncated the follow-up time at Month 21 because there were few patients remaining in the RCT arm after Month 21 and thus, the positivity assumption was unlikely to hold. This approach was adopted to emulate the end of follow-up of the target trial (Table 1). Then, to reduce variance of the weighted estimator, we calculated the stabilized IPCW weight (Austin and Stuart, 2015), which is the probability of not being censored conditional on selected baseline covariates, divided by the probability of not being censored, conditional on both baseline and time-varying covariates. The mean, standard deviation, minimum, and maximum estimated weights were used to inspect the robustness of the estimator. Estimated weights with the mean far from one—or very extreme values—are indicative of non-positivity or misspecification of the weight model (Hernán and Robins, 2006).

The treatment effects were estimated using weighted survival analysis methods. Hazard ratio (HR) and 95% CI were adopted for the population-level summary (Table 1). Specifically, we estimated the HR, using an IPTW-ATT-IPCW weighted Cox proportional hazard model and the 95% CI for the HR using the bootstrap approach (Schaubel and Wei, 2011). We also used the IPTW-ATT-IPCW weighted Kaplan-Meier method to compute OS function estimates and weighted log-rank test to compare across groups. Hence, the double weighting estimation approach targets the ATT estimand with attributes of the EF and TTF as specified in Table 1.

Missing values for covariates with a missing rate less than 30% were imputed using median (for age and time from initial diagnosis to index date) or mode (smoking history). Covariates with more than 30% of values missing (i.e., ECOG PS) were not imputed and excluded from the IPW models. We performed a sensitivity analysis by analyzing the whole follow-up period for RCT and OC arms instead of truncating them at Month 21. Also, to evaluate to what extent our estimation methods remove the potential bias on OS due to baseline confounders and intercurrent events, we performed the traditional IPTW-only method that adjusts for baseline characteristics but not intercurrent events in terms of Kaplan-Meier (K-M) estimate and HR, and compared it to our proposed method. To follow the structure of the EF, we consider this IPTW-ATT-only estimation as a supplementary analysis because it estimates an estimand different from our target estimand.

No formal hypothesis testing was conducted, and thus, no statistical significance was explicitly assessed.

R (3.6.1) was used for the analyses.

## 3 Results

### 3.1 Cohort characteristics

A total of 849 patients were in the RCT arm and 3,340 patients were in the OC arm (refer to Supplementary Table S3 for the OC cohort attrition table). Demographic and clinical characteristics of the study sample at baseline are presented in Table 2 (and in Supplementary Table S4 stratified by RCT). Statistically significant differences between the RCT and OC arms were observed in age, gender, race, ECOG PS, tumor diagnosis type (*de novo* Stage IV/recurrent disease), histology, time from initial diagnosis to index date, and treatment type. Patients in the OC arm were older, with a higher percentage of females, races other than White and Asian, recurrent disease and non-squamous histology, shorter time from initial diagnosis to index date, and less frequently treated with carboplatin plus paclitaxel/nab-paclitaxel.

The percentage of patients who switched to subsequent antineoplastic treatment, i.e., the intercurrent event of interest, was higher in the OC arm compared to the RCT arm (56.3% vs. 52.9%; Table 3) during the whole follow-up period. Among patients who switched, the median time to treatment switch was shorter in the OC arm compared to the RCT arm (5.45 vs. 6.24 months; 55.8% vs. 46.1% switched in the first 6 months). Differences in pre-specified confounders for treatment switching including age, histology, treatment type, and progression were observed. Specifically, we saw a higher percentage of switching among patients with progression events during the follow-up period in both RCT and OC arms (Table 4).

**TABLE 3:** Characteristics of intercurrent events.

	RCT	OC
Number of patients	849	3340
Median (95% CI) follow-up time, months	26.5 (19.9–28.8)	35.6 (29.4–43)
Switch to subsequent therapy (any), n (%)	449 (52.9%)	1881 (56.3%)
Median (IQR) time to switch (among patients who switched), months	6.24 (4.27–9.69)	5.45 (3.12–9.43)
Number of patients who switched prior to 6 months/Number of patients who ever switched, n (%)	207/449 (46.1)	1049/1881 (55.8)

Notes: CI, confidence interval; IQR, interquartile range; OC, observational comparator; RCT, randomized clinical trial.

**TABLE 4:** Baseline and clinical characteristics among patients who switched treatment and who did not switch treatment.

Variable	Category, n (%)	RCT		OC	
		Patients who switched treatment	Patients who did not switch treatment	Patients who switched treatment	Patients who did not switch treatment
		N = 449 (52.9%)	N = 400 (47.1%)	N = 1881 (56.3%)	N = 1459 (43.7%)
Age	<65	227 (50.6)	207 (51.9)	708 (37.6)	514 (35.2)
	65–75	179 (39.9)	143 (35.8)	717 (38.1)	551 (37.8)
	≥75	43 (9.6)	49 (12.3)	456 (24.2)	394 (27.0)
Histology	Non-squamous	251 (55.9)	258 (64.5)	1287 (68.4)	991 (67.9)
	Squamous	198 (44.1)	142 (35.5)	594 (31.6)	468 (32.1)
Treatment	Carboplatin + Pacli/Nab-pacli	287 (63.9)	281 (58.5)	1034 (55.0)	843 (57.8)
	Platinum + Pemetrexed	162 (36.1)	119 (41.5)	847 (45.0)	616 (42.2)
Progression during the follow-up^a^	Yes	390 (86.9)	230 (57.5)	1360 (72.3)	397 (27.2)
	No	59 (13.1)	170 (42.5)	521 (27.7)	1062 (72.8)

Notes: OC, observational comparator; RCT, randomized clinical trial.

^a^ Follow-up is up to switch or, in absence of switch until last activity before study end date (end date for the specific data source).

### 3.2 Main analyses

A logistic regression model was fitted to account for imbalances between the RCT and OC arms on baseline characteristics and estimate the PS. Then IPTW-ATT weights were calculated using the PS estimated from the logistic model and we excluded a small percentage of patients (0.4%) with extreme weights (weight >10) in the OC arm to avoid undesirable variability in estimates due to extremely large weights (Potter and Zheng, 2015). Supplementary Figure S2 shows the distribution of the PSs in the OC and RCT arms, which served as the basis to compute the IPTW-ATT weights. SMDs for patient variables were all below 0.1 after IPTW-ATT (Figure 1), suggesting balance achieved on the selected baseline characteristics through IPTW-ATT weighting (Austin, 2009) when trying to emulate randomization (more detail in Table 1).

Patients were artificially censored at the time of treatment switching (i.e., the intercurrent event of interest), then the censoring mechanism was modeled via a Cox regression model, and the probability of not being censored conditional on patient/clinical characteristics that were pre-specified was estimated (Table 4). The stabilized IPCW weights were calculated as the ratio of the inverse of the probability of not being censored conditional on race only and the probability of not being censored conditional on age, race, histology, and progression. Here, different from the traditional stabilized weights, race was added to both the numerator and denominator to further increase the stability of the IPCW weight (Cole and Hernán, 2008). To make a stable estimation and reduce variability, extreme weights were trimmed at the 99th percentile for the OC arm and the 98th percentile for the RCT arm. The distribution of the weights after trimming is displayed in Supplementary Table S5. The mean stabilized weights had means close to one, a necessary condition for correct model specification (Hernán and Robins, 2006).

**FIGURE 2:**
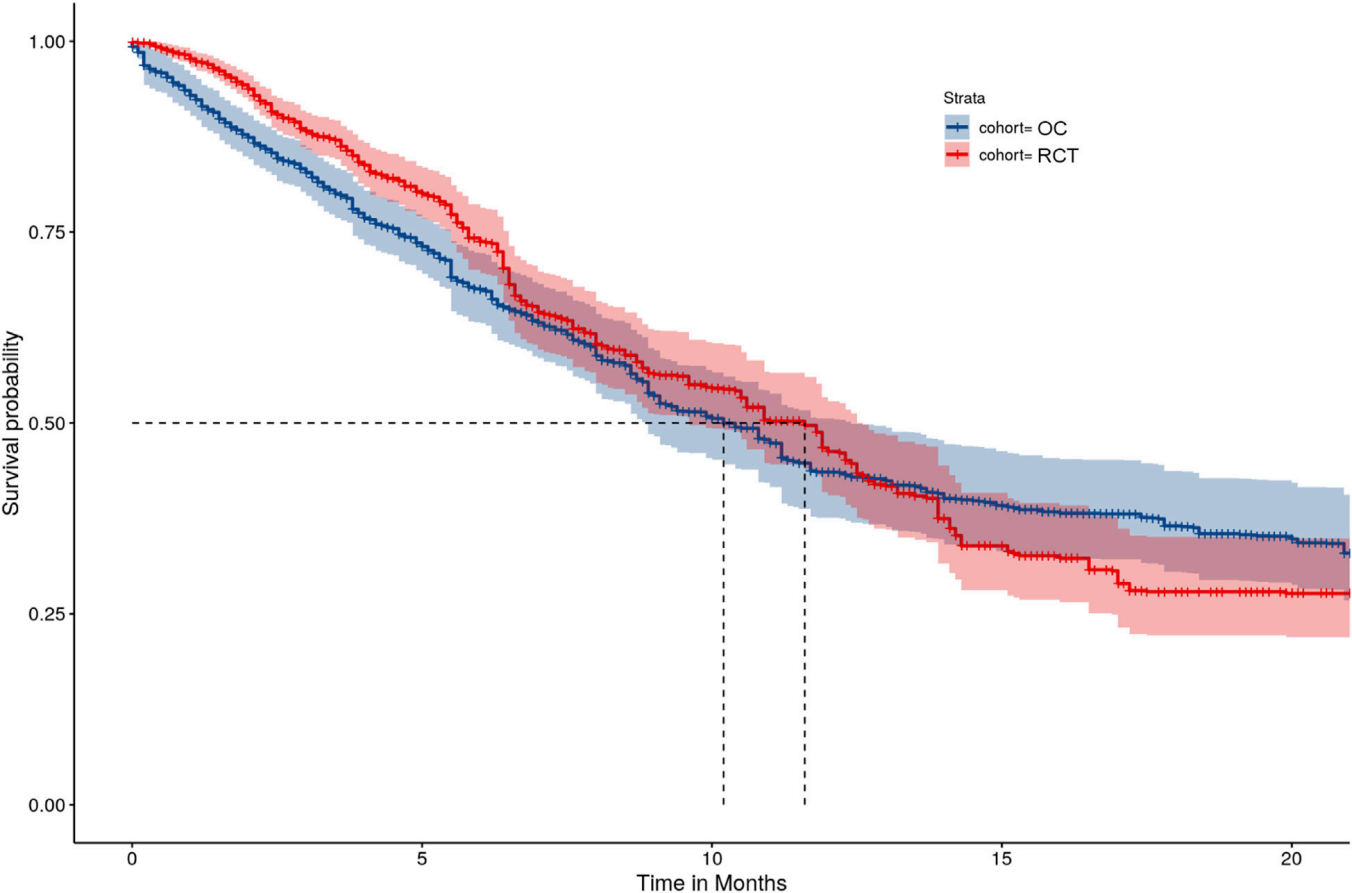
IPTW-ATT-IPCW weighted Kaplan-Meier curves.

After accounting for treatment setting assignment at baseline and treatment switching using the IPTW-ATT-IPCW method, the HR estimated from the weighted Cox model was equal to 0.94 (95% CI: [0.77, 1.13]), which suggests comparable OS between the RCT and OC arms. Weighted K-M estimates of survival functions overall were comparable (Figure 2), however, there was crossing hazard between the two arms. The two curves align well at months 7–14, while RCT performed better at months 0–6 and worse at months 15–23. The difference in median survival time between the two arms was small (9.9 months with 95% CI: [8.6, 12.3] for the OC cohort *versus* 10.9 months with 95% CI: [9.6, 12.5] for the RCT cohort). These results suggested that after accounting for imbalances of baseline characteristics and removing the confounding effects of treatment switching, patients in the OC arm had similar OS as those in the RCT arm.

### 3.3 Sensitivity analyses

**FIGURE 3:**
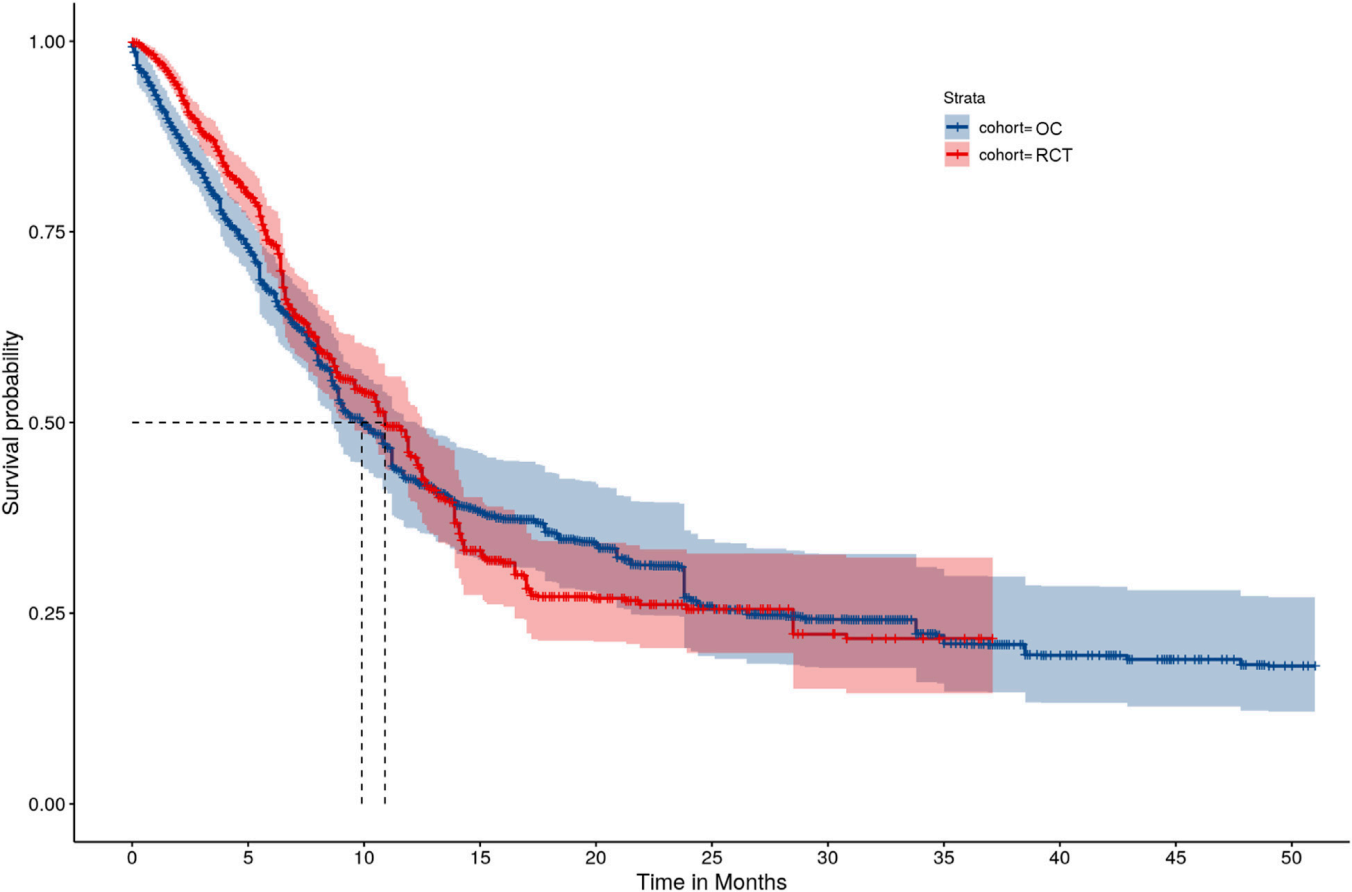
IPTW-ATT-IPCW weighted Kaplan-Meier survival function estimates without truncating the follow-up time.

A sensitivity analysis was performed to analyze the entire follow-up period (i.e., no truncation at 21 months) for the RCT and OC arms. The HR was 0.93 (95% CI: [0.77, 1.13]), which was similar to the primary analysis results. However, there were wider confidence intervals for K-M curves after month 21 for both the RCT and OC arms due to the low number of events (Figure 3).

### 3.4 Supplemental analyses

**FIGURE 4:**
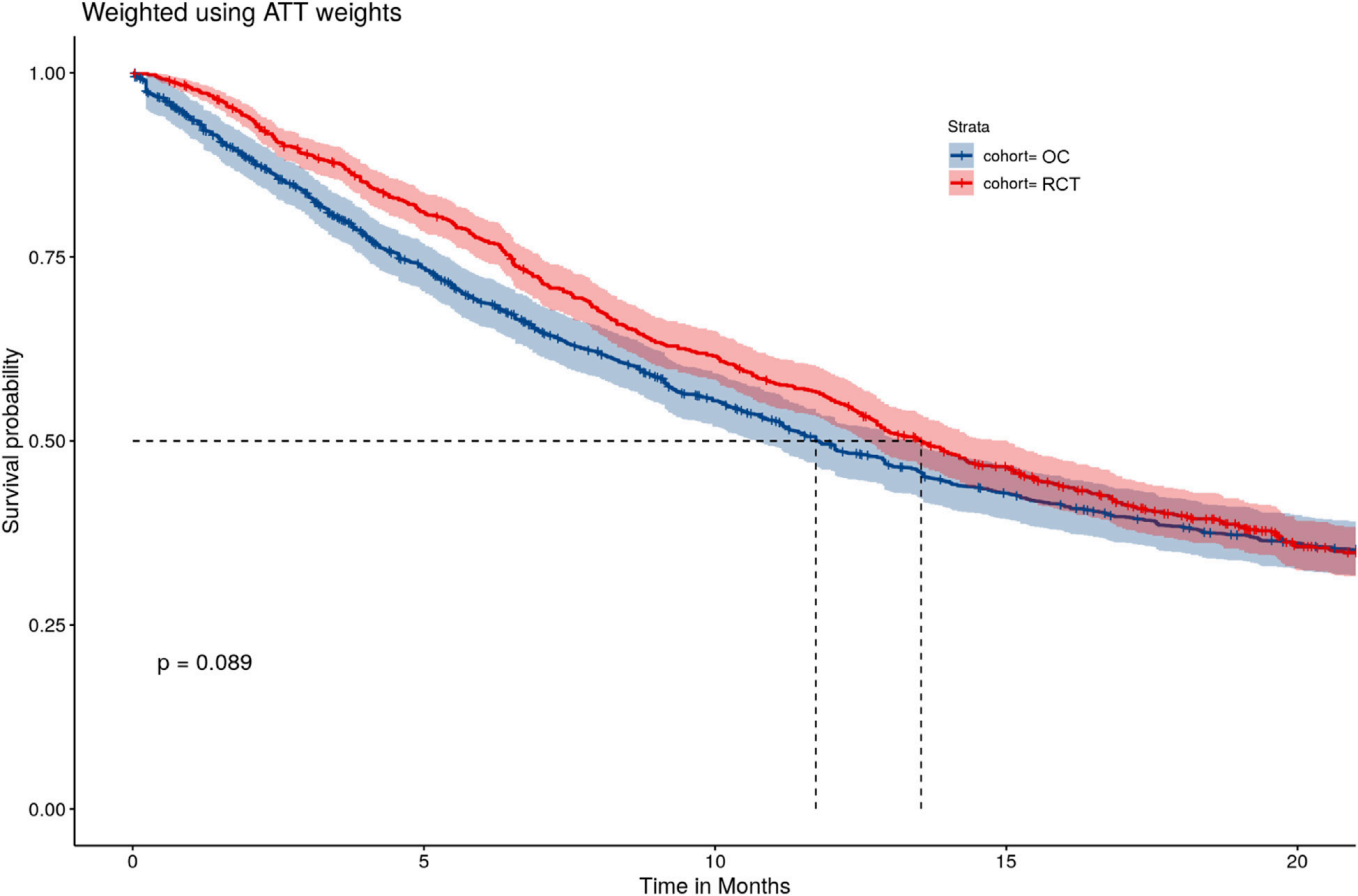
IPTW-ATT weighted Kaplan-Meier curves.

In a supplemental analysis, we performed an IPTW-ATT-only analysis that adjusted for baseline characteristics only by IPTW-ATT weighting but without IPCW. This is a commonly used method in analyses of external control arms, resulting in a different estimand compared to the primary analysis. Although the HR was similar to the primary analysis (0.92, 95% CI: [0.81, 1.05]), there was a larger discrepancy in K-M estimates between the RCT and OC arms, especially during Months 6 and 14, compared to the primary analysis (Figure 4).

## 4 Discussion

In this study, we applied the EF and TTF to define a precise scientific question in comparative-effectiveness research. As a case study to illustrate how to apply the EF and TTF when designing an external control study using observational data, we conducted a retrospective cohort study to compare OS among patients with metastatic NSCLC exposed to front-line chemotherapy in RCTs *versus* routine clinical practice settings, while accounting for differences in subsequent treatments between these settings. To achieve this objective, we pooled clinical trial patients from the control arms of three RCTs (IMPOWER 130, 131, and 132) and derived an OC cohort from de-identified EHR data obtained from routine clinical practice. OS was compared between the two arms, assuming a hypothetical scenario wherein patients in neither setting received subsequent therapy after the first-line chemotherapy. We found no relevant difference in OS between the two arms. Hence, when accounting for baseline confounding as well as differences in patterns of subsequent treatments in clinical trial and routine clinical practice care patients, the long-term outcome of first-line treatment for patients with metastatic NSCLC is similar despite the lack of full trial entry criteria implementation.

Our approach attempts to clarify the causal contrast of interest by combining elements of the EF and TTF. The EF and TTF serve complementary purposes in answering the scientific question. As formulated by Hernán and Robins, the TTF ensures that an appropriate comparative study is designed to help estimate the causal effect from the observed data (Hernán and Robins, 2016). While the causal contrast can be specified within the TTF, the EF adds clarity to the causal contrast through the explicit consideration of intercurrent events (i.e., events occurring post-baseline that can affect the assessment of treatment effects). Combined, the EF and TTF improves transparency in the: 1) target of estimation (causal contrast), 2) assumptions and data needed to identify the causal contrast, and 3) limitations of available data.

To our knowledge, there are a limited number of studies that combine the EF and TTF. Recently, Hampson et al. combined the EF and TTF using routine clinical care data to generate an external control arm (Hampson et al., 2023). The approach described in our study adds to the limited number of use cases by accounting for a scenario where patterns of subsequent treatments are different between the sources of clinical trials and routine clinical care. We anticipate that many researchers will likely encounter this scenario in applications involving real-world external controls. Our study, unlike other studies, also illustrates the iterative nature of specifying an estimand. In practice, such iteration allows a comprehensive and transparent dialogue among stakeholders to reach a consensus on the scientific question and its tractability given the available data (i.e., discern the identifiability of the estimand).

Strengths of this study include the combination of the EF and TTF, its large sample size, extensive follow-up, and its high proportion of patients with an event of interest. In addition, to mitigate possible sources of bias due to heterogeneity from comparing the RCT and OC arms, we emulated randomization with IPTW. Furthermore, the real-world data source we selected reports key variables with high accuracy and clinical relevance. For example, the composite real-world mortality endpoint was previously validated using the National Death Index, and the real-world disease progression endpoint, although following a clinician-anchored approach supported by radiology report data, was previously found to be comparable to trial RECIST-based disease progression (Griffith et al., 2019; Zhang et al., 2021; Mhatre et al., 2023). Lastly, model diagnostics indicated that the weights from the IPTW-ATT and IPCW induced balance in the measured baseline and post-baseline confounders.

There are notable limitations with this study. First, because data were pooled from disparate sources, full information was not available for all possible confounders. For example, there was limited capture of comorbidities, sites of metastasis, and smoking status within the OC arm compared to the RCT arm. The assumption of no unmeasured confounders underlies both IPCW (i.e., baseline as well as time-varying covariates jointly predicting treatment switch and outcome (Howe et al., 2011)) as well as IPTW (i.e., baseline covariates jointly predicting treatment setting and outcome). About 43% (Table 2) of the patients in routine clinical care included in our study had missing ECOG PS at the start of front-line therapy, some of whom may have had an ECOG PS value above 1. For context, among adults with NSCLC who received first-line chemotherapy in the real-world setting, 13.6% had an ECOG PS greater than 1 (Supplementary Table S3). A second limitation was that the definition of time-zero differed across the RCT and OC arms. Time-zero was the date of randomization in the clinical trials compared to the date of treatment initiation in the routine clinical practice cohort. The impact is believed to be small given that typically, treatment was initiated within a few days post-randomization. A third limitation is that patients in the IMpower trials were global while patients in the OC arm were from the United States only. Although we account for potential patient confounders in our models, there could be residual confounding effects due to regional differences. A fourth limitation was that we pooled data from the control arms of the RCTs and hence assumed negligible heterogeneity in outcomes among the three clinical-trial cohorts. However, we believe trial heterogeneity posed little bias risk to our study because the three trials were conducted by the same sponsor and had similar visit schedules, data quality monitoring, and survival estimates (Supplementary Figure S1). As a final limitation, this work does not present guidelines regarding size and power because formal hypothesis testing was not conducted during the study. A proper power analysis would need to specify and model the impact of the (time-varying) confounders on the effect size. There are limited examples applying time-varying covariate weighting in external control analyses, and guidelines on how to compute sample size are needed. Future work should aim to establish size and power guidelines to ensure quality and meaningful inferences from these types of analyses.

## 5 Conclusion

In conclusion, this study showed that combining the EF and TTF approaches can improve the rigor in the design and analysis of comparative effectiveness studies, including retrospective observational studies. The EF approach alone does not suffice in specifying a study design, and the TTF alone can leave ambiguity in the inferential target. The combination of the two frameworks should be considered more often by researchers.

## Data Availability

The datasets presented in this article are not readily available because the real-world data that support the findings of this study have been originated by Flatiron Health, Inc. Requests for data sharing by license or by permission for the specific purpose of replicating results in this manuscript can be submitted to publicationsdataaccess@flatiron.com. For eligible clinical trials, qualified researchers may request access to individual patient-level clinical data for each separate study through a data request platform. At the time of writing, this request platform is Vivli. https://vivli.org/ourmember/roche/. For up to date details on Roche’ Global Policy on the Sharing of Clinical Information and how to request access to related clinical study documents, see here: https://go.roche.com/data_sharing. Anonymized records for individual patients across more than one data source external to Roche cannot, and should not, be linked due to a potential increase in risk of patient re-identification.
