# Supplementary material for: Applying the estimand and target trial frameworks to external control analyses using observational data: a case study in the solid tumor setting
Source: Front Pharmacol. 2024 Jan 26;15:1223858. doi: 10.3389/fphar.2024.1223858 (PMC10853363; doi:10.3389/fphar.2024.1223858)
Supplement: Supplementary file 1 [file DataSheet1.DOCX]

***Supplementary Material***

Applying the Estimand and Target Trial frameworks to external control analyses using observational data: a case study in the solid tumor setting

**Letizia Polito^*†^, Qixing Liang^†^, Navdeep Pal, Philani Mpofu, Ahmed Sawas, Olivier Humblet, Kaspar Rufibach, Dominik Heinzmann**

*** Correspondence:** Corresponding Author: [letizia.polito@roche.com](mailto:letizia.polito@roche.com)

†These authors contributed equally to this work and share first authorship

# Supplementary Figures and Tables

## Supplementary Figures


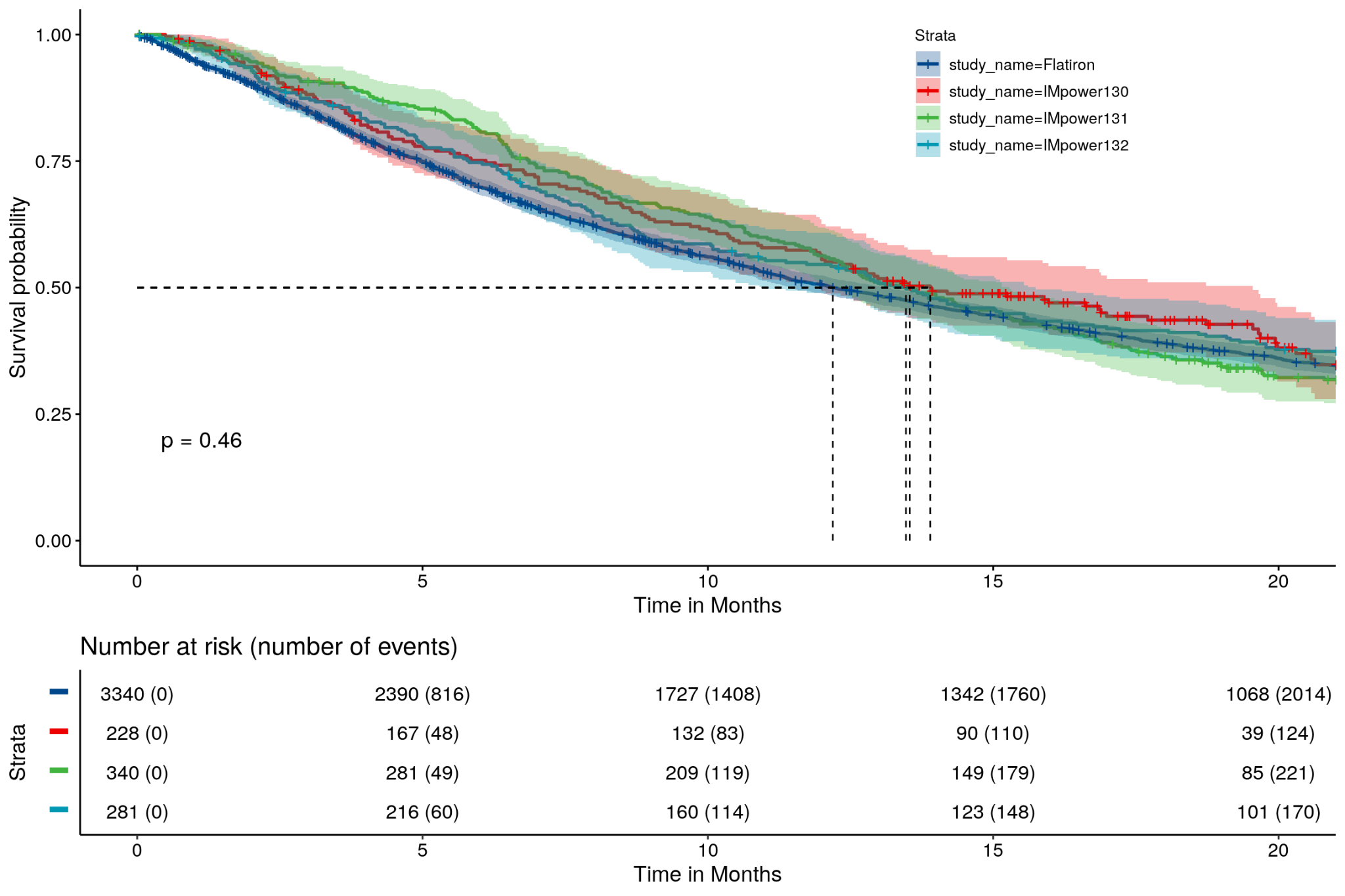
**Supplementary Figure 1.** Kaplan-Meier plots showing the comparison of overall survival between trials were considered separately.


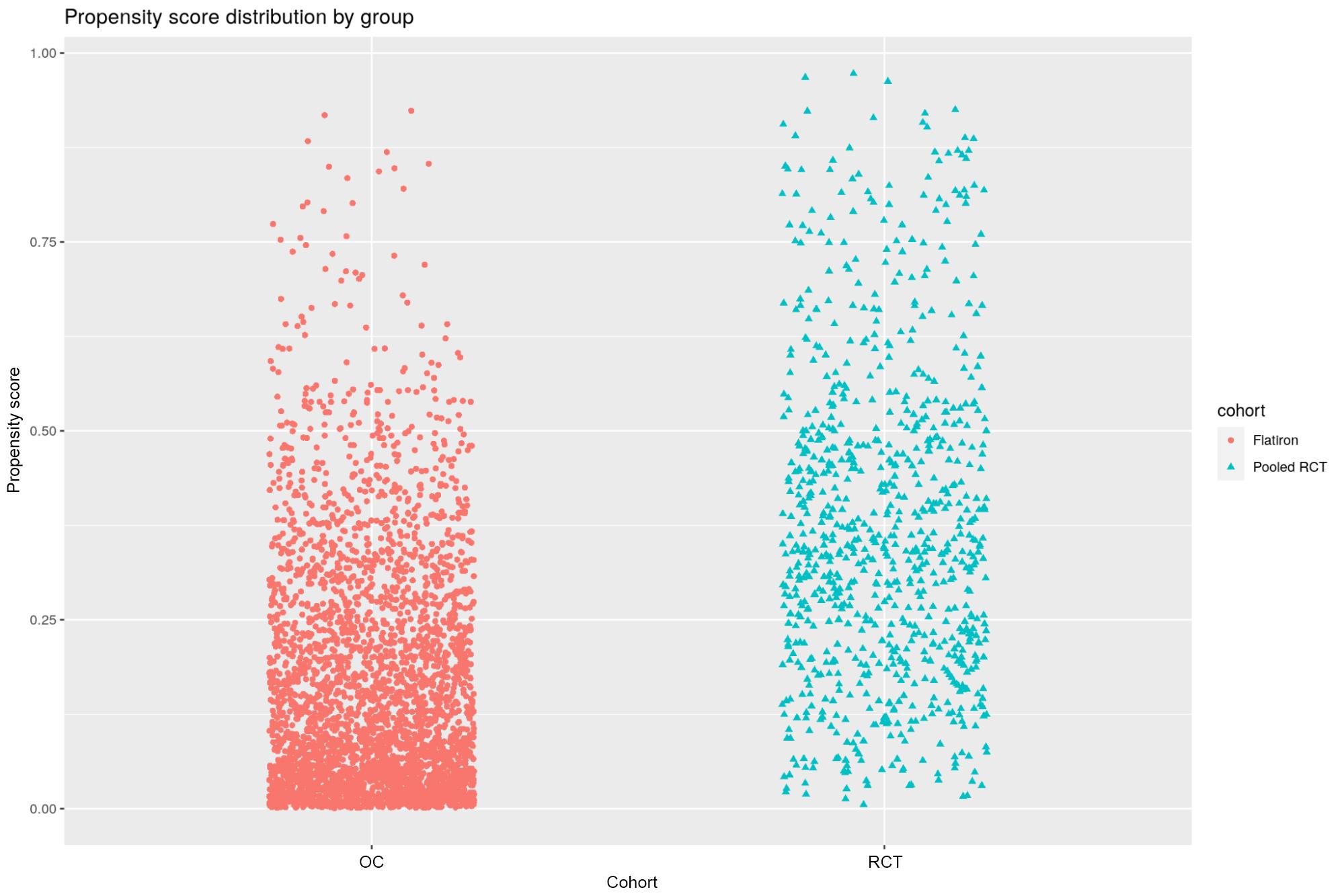


**Supplementary Figure 2.** Propensity score distribution used to compute IPTW-ATT weights. OC = observational comparator. RCT = randomized clinical trial (pooled).

##

## Supplementary Tables

**Supplementary Table 1.** Alignment of study time window between RCT and OC arms.

| **Study** | **Enrollment start date** | **Enrollment end date** | **Primary completion date** |
| --- | --- | --- | --- |
| IMpower130 | Apr 16, 2015 | Feb 13, 2017 | Mar 15, 2018 |
| IMpower131 | Jun 11, 2015 | Mar 28, 2017 | Oct 3, 2018 |
| IMpower132 | Apr 7, 2016 | May 31, 2017 | July 18, 2019 |
| **Pooled RCT arm** | **Apr 16, 2015** | **May 31, 2017** | **July 18, 2019** |
| **OC arm ^a^** | **Apr 16, 2015 ^b^** | **May 31, 2017** | **July 18, 2019** |

Notes: OC = observational comparator; RCT = randomized clinical trial.

^a^ OC arm covers the entire enrollment and follow-up period of the three trials

^b^ Treatment initiation date

**Supplementary Table 2.** Detailed inclusion/exclusion criteria.

| **IMpower130** | **IMpower131** | **IMpower132** | **OC** |
| --- | --- | --- | --- |
| ***Inclusion Criteria*** | | | |
| *Signed Informed Consent Form* | *Signed Informed Consent Form* | *Signed Informed Consent Form* | *Not implemented*  *(Not relevant)* |
| *Male or female, 18 years of age or older* | *Male or female, 18 years of age or older* | *Male or female, 18 years of age or older* | *Implemented*  *(Age at start of treatment)* |
| *ECOG performance status of 0 or 1* | *ECOG performance status of 0 or 1* | *ECOG performance status of 0 or 1* | *Implemented ECOG PS 0 or 1 or missing*  *(The closest ECOG value within a -30 to +7 window around 1L start date will be used, taking the highest if multiple observations occur on same day)* |
| *Histologically or cytologically confirmed, Stage IV* ***non-squamous*** *NSCLC* | *Histologically or cytologically confirmed, Stage IV* ***squamous*** *NSCLC* | *Histologically or cytologically confirmed, Stage IV* ***non-squamous*** *NSCLC*   - *Patients with tumors of mixed non-small cell histology (i.e., squamous and non- squamous) were eligible if the major histological component appeared to be non-squamous.* | *Implemented*  *(either squamous or non-squamous)* |
| *No prior treatment for Stage IV* ***non-squamous*** *NSCLC* | *No prior treatment for Stage IV* ***squamous*** *NSCLC* | *No prior treatment for Stage IV* ***non-squamous*** *NSCLC* | *Implemented*  *(Patients with structured activity within 90 days of advanced diagnosis)* |
| *Patients who have received prior neo-adjuvant, adjuvant chemotherapy, or chemoradiotherapy with curative intent for non-metastatic disease must have experienced a treatment-free interval of at least 6 months from randomization since the last chemotherapy or chemoradiotherapy cycle.* | *Patients who have received prior neo-adjuvant, adjuvant chemotherapy,* ***radiotherapy****, or chemoradiotherapy with curative intent for non-metastatic disease must have experienced a treatment-free interval of at least 6 months from randomization since the last chemotherapy, radiotherapy, or chemoradiotherapy.* | *Patients who had received prior neo-adjuvant, adjuvant chemotherapy,* ***radiotherapy****, or chemoradiotherapy with curative intent for non-metastatic disease must have experienced a treatment-free interval of at least 6 months from randomization since the last dose of chemotherapy and/or radiotherapy* | *Implemented*  *(Patients with documented antineoplastic medications 6 months prior to 1L start date, per medication administration and enhanced orals tables)* |
| *Patients with a history of treated asymptomatic CNS metastases are eligible, provided they meet all of the following criteria:*   - *Only supratentorial and cerebellar metastases allowed (i.e., no metastases to midbrain, pons, medulla or spinal cord)* - *No ongoing requirement for corticosteroids as therapy for CNS disease* - *No stereotactic radiation within 7 days or whole-brain radiation within 14 days prior to randomization* - *No evidence of interim progression between the completion of CNS-directed therapy and the screening radiographic study* | *Patients with a history of treated asymptomatic CNS metastases are eligible, provided they meet all of the following criteria:*   - *Only supratentorial and cerebellar metastases allowed (i.e., no metastases to midbrain, pons, medulla, or spinal cord)* - *No ongoing requirement for corticosteroids as therapy for CNS disease* - *No stereotactic radiation within 7 days or whole-brain radiation within 14 days prior to randomization* - *No evidence of interim progression between the completion of CNS-directed therapy and the screening radiographic study.* | *Patients with a history of treated asymptomatic CNS metastases were eligible, provided they met all of the following criteria:*   - *Only supratentorial and cerebellar metastases were allowed (i.e., no metastases to midbrain, pons, medulla, or spinal cord)* - *No ongoing requirement for corticosteroids as therapy for CNS disease* - *No stereotactic radiation within 7 days or whole-brain radiation within 14 days prior to randomization* - *No evidence of interim progression between the completion of CNS-directed therapy and the screening radiographic study* | *Not implemented*  *(Not in real-world database)* |
| *NA* | *NA* | *Patients were to submit a pre-treatment tumor tissue sample (if available). If tumor tissue was not available (e.g., depleted for prior diagnostic testing), patients were still eligible.* | *Not implemented* |
| ***Known PD-L1 tumor status*** *as determined by an IHC assay performed by a central laboratory on previously obtained archival tumor tissue or tissue obtained from a biopsy at screening* | ***Known PD-L1 tumor status*** *as determined by an IHC assay performed by a central laboratory on previously obtained archival tumor tissue or tissue obtained from a biopsy at screening.* | ***Known tumor PD-L1*** *expression status as determined by an IHC assay from other clinical studies* | *Not implemented*  *(Include all patients regardless of PD-L1 status)* |
| *Measurable disease, as defined by RECIST v1.1* | *Measurable disease, as defined by RECIST v1.1* | *Measurable disease, as defined by RECIST v1.1* | *Not implemented*  *(Not in real-world database)* |
| *Adequate hematologic and end-organ function, defined by the following laboratory results obtained within 14 days prior to randomization:* | *Adequate hematologic and end-organ function, defined by the following laboratory results obtained within 14 days prior to randomization:* | *Adequate hematologic and end-organ function, defined by the following laboratory results obtained within 14 days prior to randomization:* | *See the following 8 rows.*  *Adequate hematological and end-organ function at start of index therapy. For all of the above labs, the closest result in the -28 to 0 window around 1L start will be used. Patients with no recorded results for a specific lab in that time window will not be excluded.* |
| - *ANC ≥1500 cells/μL without granulocyte colony-stimulating factor support* | - *ANC ≥1500 cells/μL without granulocyte colony-stimulating factor support* | - *ANC ≥1500 cells/μL without granulocyte colony-stimulating factor support* | *Implemented* |
| - *Lymphocyte count ≥500 cells/μL* | - *Lymphocyte count ≥500 cells/μL* | - *Lymphocyte count ≥500 cells/μL* | *Implemented* |
| - *Platelet count ≥100,000 cells/μL without transfusion* | - *Platelet count ≥100,000 cells/μL without transfusion* | - *Platelet count ≥100,000 cells/μL without transfusion* | *Implemented* |
| - *Hemoglobin ≥9.0 g/dL* | - *Hemoglobin ≥9.0 g/dL* | - *Hemoglobin ≥9.0 g/dL* | *Implemented* |
| - *INR or aPTT ≤1.5 x ULN* | - *INR or aPTT ≤1.5 x ULN* | - *INR or aPTT ≤1.5 x ULN* | *Not Implemented*  *(Highly missing)* |
| - *AST, ALT, and alkaline phosphatase ≤2.5 x ULN with the following exceptions:*   *Patients with documented liver metastases: AST and/or ALT ≤5 x ULN;*  *Patients with documented liver or bone metastases: alkaline phosphatase ≤5 x ULN* | - *AST, ALT, and alkaline phosphatase ≤2.5 x ULN with the following exceptions:*   *Patients with documented liver metastases: AST and/or ALT ≤5 x ULN;*  *Patients with documented liver or bone metastases: alkaline phosphatase ≤5 x ULN* | - *AST, ALT, and alkaline phosphatase ≤2.5 x ULN with the following exceptions:*   *Patients with documented liver metastases: AST and/or ALT ≤5 x ULN;*  *Patients with documented liver or bone metastases: alkaline phosphatase ≤5 x ULN* | *Implemented*  *(Same, though will not consider the exceptions)* |
| - *Serum bilirubin ≤1.5 x ULN* | - *Serum bilirubin ≤1.5 x ULN* | - *Serum bilirubin ≤****1.25*** *x ULN* | *Implemented*  *(≤1.5 x ULN)* |
| *N/A* | - *Calculated CrCl ≥45 mL/min, or if using cisplatin, calculated CrCl ≥60 mL/min* | - *Calculated creatinine clearance (CrCl) ≥45 mL/min, or if using cisplatin, calculated CrCl ≥60 mL/min* | *Not implemented* |
| *NA* | *NA* | *For patients enrolled in the extended China enrollment phase: current resident of*  *mainland China, Hong Kong, or Taiwan and of Chinese ancestry* | *Not implemented*  *(Not in real-world database)* |
| *For female patients of childbearing potential and male patients with partners of childbearing potential, agreement (by patient and/or partner) to use a highly effective form(s) of contraception that results in a low failure rate [< 1% per year] when used consistently and correctly, and to continue its use for* ***90 days*** *after the last dose of atezolizumab or for 30 days after the last dose of nab-paclitaxel, whichever is later. For male patients with female partners of childbearing potential, agreement (by patient and/or partner) to use a highly effective form(s) of contraception that results in a low failure rate [< 1% per year] when used consistently and correctly, and to continue its use for 90 days after the last dose of atezolizumab or for 6 months after the last dose of nab-paclitaxel, whichever is later. Such methods include: combined(estrogen and progestogen containing) hormonal contraception, progestogen-only hormonal contraception associated with inhibition of ovulation together with another additional barrier method always containing a spermicide, intrauterine device (IUD), intrauterine hormone -releasing system (IUS), bilateral tubal occlusion or vasectomized partner (on the understanding that this is the only one partner during the whole study duration), and sexual abstinence. Male patients should not donate sperm during this study and for at least 6 months after the last dose of nab-paclitaxel.* | *For female patients of childbearing potential, agreement (by patient and/or partner) to use a highly effective form(s) of contraception that results in a low failure rate (< 1% per year) when used consistently and correctly, and to continue its use for* ***5 months*** *after the last dose of atezolizumab, for 30 days after the last dose of nab-paclitaxel, or for 6 months after the last dose of paclitaxel, whichever is later. Women must refrain from donating eggs during this same period. For male patients with female partners of childbearing potential, agreement (by patient and/or partner) to use a highly effective form(s) of contraception that results in a low failure rate (< 1% per year) when used consistently and correctly, and to continue its use for 6 months after the last dose of nab-paclitaxel, paclitaxel, and/or carboplatin. Such methods include combined (estrogen and progestogen containing) hormonal contraception, progestogen-only hormonal contraception associated with inhibition of ovulation together with another additional barrier method always containing a spermicide, intrauterine device (IUD), intrauterine hormone-releasing system (IUS), bilateral tubal occlusion or vasectomized partner (on the understanding that this is the only one partner during the whole study duration), and sexual abstinence. Men must refrain from donating sperm during the study and for 6 months after the last dose of nab-paclitaxel, paclitaxel, and/or carboplatin, whichever is latest.* | *For women of childbearing potential: agreement to remain abstinent (refrain from heterosexual intercourse) or use contraceptive methods that result in a failure rate of 1% per year during the treatment period and for at least 5 months after the last dose of atezolizumab or 6 months after the last dose of cisplatin.*  *A woman was considered to be of childbearing potential if she was postmenarcheal, had not reached a postmenopausal state ( 12 continuous months of amenorrhea with no identified cause other than menopause), and had not undergone surgical sterilization (removal of ovaries and/or uterus).*  *Examples of non-hormonal contraceptive methods with a failure rate of*  *< 1% per year included bilateral tubal ligation, male sterilization, established, proper use of hormonal contraceptives that inhibit ovulation, hormone-releasing intrauterine devices, and copper intrauterine devices.*  *The reliability of sexual abstinence was to be evaluated in relation to the duration of the clinical trial and the preferred and usual lifestyle of the patient. Periodic abstinence (e.g., calendar, ovulation, symptothermal, or postovulation methods) and withdrawal were not acceptable methods of contraception.*  *For men: agreement to remain abstinent (refrain from heterosexual intercourse) or use contraceptive measures and agreement to refrain from donating sperm, as defined below:*  *With partners of childbearing potential, men were to remain abstinent or use a condom plus an additional contraceptive method that together resulted in a failure rate of 1% per year during the chemotherapy treatment period and for at least 6 months after the last dose of chemotherapy.*  *With pregnant partners, men were to remain abstinent or use a condom during the chemotherapy treatment period and for at least 6 months after the last dose of chemotherapy.*  *The reliability of sexual abstinence was to be evaluated in relation to the duration of the clinical trial and the preferred and usual lifestyle of the patient. Periodic abstinence (e.g., calendar, ovulation, symptothermal, or postovulation methods) and withdrawal were not acceptable methods of contraception.* | *Not implemented*  *(Not in real-world database)* |
| *Oral contraception should always be combined with an additional contraceptive method because of a potential interaction with the study drug. The same rules are valid for male patients involved in this clinical study if they have a partner of childbirth potential. Male patients must always use a condom.* | *Oral contraception should always be combined with an additional contraceptive method because of a potential interaction with the study drug. The same rules are valid for male patients involved in this clinical trial if they have a partner of childbearing potential. Male patients must always use a condom.* |  | *Not implemented*  *(Not in real-world database)* |
| *Women who are not postmenopausal (≥12 months of non-therapy-induced amenorrhea) or surgically sterile must have a negative serum pregnancy test result within 14 days prior to initiation of study drug.* | *Women who are not postmenopausal (≥12 months of non-therapy induced amenorrhea) or surgically sterile must have a negative serum pregnancy test result within 14 days prior to initiation of study drug.* |  | *Not implemented*  *(Not in real-world database)* |
|  | | | |
| ***Exclusion*** | | | |
| ***Cancer-Specific Exclusions*** | | | |
| *Known tumor PD-L1 expression status as determined by an IHC assay from other clinical studies* | *Known tumor PD-L1 expression status as determined by an IHC assay from other clinical studies* | *Known tumor PD-L1 expression status as determined by an IHC assay from other clinical studies* | *Not implemented* |
| *N/A* | *N/A* | *Patients with a sensitizing mutation in the EGFR gene or an ALK fusion oncogene* | *Implemented* |
| *Active or untreated CNS metastases as determined by CT or MRI evaluation during screening and prior radiographic assessments* | *Active or untreated CNS metastases as determined by CT or MRI evaluation during screening and prior radiographic assessments* | *Active or untreated CNS metastases as determined by CT or MRI evaluation during screening and prior radiographic assessments* | *Not implemented*  *(Not in real-world database)* |
| *Spinal cord compression not definitively treated with surgery and/or radiation or previously diagnosed and treated spinal cord compression without evidence that disease has been clinically stable for >2 weeks prior to randomization* | *Spinal cord compression not definitively treated with surgery and/or radiation or previously diagnosed and treated spinal cord compression without evidence that disease has been clinically stable for >2 weeks prior to randomization* | *Spinal cord compression not definitively treated with surgery and/or radiation or previously diagnosed and treated spinal cord compression without evidence that disease had been clinically stable for* ***≥*** *2 weeks prior to randomization* | *Not implemented*  *(Not in real-world database)* |
| *Leptomeningeal disease* | *Leptomeningeal disease* | *Leptomeningeal disease* | *Not implemented*  *(Not in real-world database)* |
| *Uncontrolled tumor-related pain* | *Uncontrolled tumor-related pain* | *Uncontrolled tumor-related pain* | *Not implemented*  *(Not in real-world database)* |
| *Uncontrolled pleural effusion, pericardial effusion, or ascites requiring recurrent drainage procedures (once monthly or more frequently)* | *Uncontrolled pleural effusion, pericardial effusion, or ascites requiring recurrent drainage procedures (once monthly or more frequently)* | *Uncontrolled pleural effusion, pericardial effusion, or ascites requiring recurrent drainage procedures (once monthly or more frequently)* | *Not implemented*  *(Not in real-world database)* |
| *Uncontrolled or symptomatic hypercalcemia (>1.5 mmol/L ionized calcium or Ca >12 mg/dL or corrected serum calcium >ULN)* | *Uncontrolled or symptomatic hypercalcemia (>1.5 mmol/L ionized calcium or Ca >12 mg/dL or corrected serum calcium >ULN)* | *Uncontrolled or symptomatic hypercalcemia (>1.5 mmol/L ionized calcium or Ca >12 mg/dL or corrected serum calcium >ULN)* | *Implemented*  *(Same)* |
| *Malignancies other than NSCLC within 5 years prior to randomization, with the exception of those with a negligible risk of metastasis or death treated with expected curative outcome* | *Malignancies other than NSCLC within 5 years prior to randomization, with the exception of those with a negligible risk of metastasis or death treated with expected curative outcome* | *Malignancies other than NSCLC within 5 years prior to randomization, with the exception of those with a negligible risk of metastasis or death treated with expected curative outcome* | *Not implemented*  *(Not in real-world database)* |
|  | | | |
| ***General Medical Exclusions*** | | | |
| *Women who are pregnant, lactating, or intending to become pregnant during the study* | *Women who are pregnant, lactating or intending to become pregnant during the study* | *Women who are pregnant, lactating or intending to become pregnant during the study* | *Not implemented*  *(Not in real-world database)* |
| *History of severe allergic, anaphylactic, or other hypersensitivity reactions to chimeric or humanized antibodies or fusion proteins* | *History of severe allergic, anaphylactic, or other hypersensitivity reactions to chimeric or humanized antibodies or fusion proteins* | *History of severe allergic, anaphylactic, or other hypersensitivity reactions to chimeric or humanized antibodies or fusion proteins* | *Not implemented*  *(Not in real-world database)* |
| *Known hypersensitivity or allergy to biopharmaceuticals produced in Chinese hamster ovary cells or any component of the atezolizumab formulation* | *Known hypersensitivity or allergy to biopharmaceuticals produced in Chinese hamster ovary cells or any component of the atezolizumab formulation* | *Known hypersensitivity or allergy to biopharmaceuticals produced in Chinese hamster ovary cells or any component of the atezolizumab formulation* | *Not implemented*  *(Not in real-world database)* |
| *History of autoimmune disease* | *History of autoimmune disease* | *History of autoimmune disease* | *Not implemented*  *(Not in real-world database)* |
| *History of idiopathic pulmonary fibrosis, organizing pneumonia (e.g., bronchiolitis obliterans), drug-induced pneumonitis, idiopathic pneumonitis, or evidence of active pneumonitis on screening chest CT scan* | *History of idiopathic pulmonary fibrosis, organizing pneumonia (e.g., bronchiolitis obliterans), drug-induced pneumonitis, idiopathic pneumonitis, or evidence of active pneumonitis on screening chest CT scan* | *History of idiopathic pulmonary fibrosis, organizing pneumonia (e.g., bronchiolitis obliterans), drug-induced pneumonitis, idiopathic pneumonitis, or evidence of active pneumonitis on screening chest CT scan* | *Not implemented*  *(Not in real-world database)* |
| *Positive test for HIV (prior to randomization)* | *Positive test for HIV (prior to randomization)* | *Positive test for HIV (prior to randomization)* | *Implemented*  *(Same)* |
| *Patients with active hepatitis B (chronic or acute; defined as having a positive HBsAg test at screening) or hepatitis C*   - *Patients with past HBV infection or resolved HBV infection (defined as the presence of hepatitis B core antibody and absence of HBsAg) are eligible. HBV DNA test must be performed in these patients prior to randomization.* - *Patients positive for HCV antibody are eligible only if PCR is negative for HCV RNA.* | *Patients with active hepatitis B (chronic or acute; defined as having a positive HBsAg test at screening) or hepatitis C*   - *Patients with past HBV infection or resolved HBV infection (defined as the presence of hepatitis B core antibody and absence of HBsAg) are eligible. HBV DNA test must be performed in these patients prior to randomization.* - *Patients positive for HCV antibody are eligible only if PCR is negative for HCV RNA.* | *Patients with active hepatitis B (chronic or acute; defined as having a positive HBsAg test at screening) or hepatitis C*   - *Patients with past HBV infection or resolved HBV infection (defined as the presence of HBcAb and absence of HBsAg) were eligible only if they were negative for HBV DNA.* - *Patients positive for HCV antibody were eligible only if PCR was negative for HCV RNA.* | *Implemented*  *(Same)* |
| *Active tuberculosis* | *Active tuberculosis* | *Active tuberculosis* | *Not implemented*  *(Not in real-world database)* |
| *Severe infections within 4 weeks prior to randomization, including but not limited to hospitalization for complications of infection, bacteremia, or severe pneumonia* | *Severe infections within 4 weeks prior to randomization, including, but not limited to, hospitalization for complications of infection, bacteremia, or severe pneumonia* | *Severe infections within 4 weeks prior to randomization, including but not limited to*  *hospitalization for complications of infection, bacteremia, or severe pneumonia* | *Not implemented*  *(Not in real-world database)* |
| *Received therapeutic oral or IV antibiotics within 2 weeks prior to randomization* | *Received therapeutic oral or IV antibiotics within 2 weeks prior to randomization* | *Received therapeutic oral or IV antibiotics within 2 weeks prior to randomization* | *Implemented*  *(Likely low completeness. Using the real-world database, it is not possible to distinguish between therapeutic and prophylactic antibiotics, leading to potential for misclassification if this criteria were applied.)* |
| *Significant cardiovascular disease, such as New York Heart Association cardiac disease (Class II or greater), myocardial infarction within 3 months prior to randomization, unstable arrhythmias, or unstable angina* | *Significant cardiovascular disease, such as New York Heart Association cardiac disease (Class II or greater), myocardial infarction, or* ***cerebrovascular accident*** *within the 3 months prior to randomization, unstable arrhythmias, or unstable angina* | *Significant cardiovascular disease, such as New York Heart Association cardiac disease (Class II or greater), myocardial infarction, or* ***cerebrovascular*** ***accident*** *within 3 months prior to randomization, unstable arrhythmias, or unstable angina* | *Not implemented*  *(Not in real-world database)* |
| *Major surgical procedure other than for diagnosis within 28 days prior to randomization or anticipation of need for a major surgical procedure during the course of the study* | *Major surgical procedure other than for diagnosis within 28 days prior to randomization or anticipation of need for a major surgical procedure during the course of the study* | *Major surgical procedure other than for diagnosis within 28 days prior to randomization or anticipation of need for a major surgical procedure during the course of the study* | *Not implemented*  *(Not in real-world database)* |
| *Prior allogeneic bone marrow transplantation or solid organ transplant* | *Prior allogeneic bone marrow transplantation or solid organ transplant* | *Prior allogeneic bone marrow transplantation or solid organ transplant* | *Not implemented*  *(Not in real-world database)* |
| *Administration of a live, attenuated vaccine within 4 weeks before randomization or anticipation that such a live attenuated vaccine will be required during the study* | *Administration of a live, attenuated vaccine within 4 weeks before randomization or anticipation that such a live attenuated vaccine will be required during the study* | *Administration of a live attenuated vaccine within 4 weeks before randomization or*  *anticipation that such a live attenuated vaccine would be required during the study* | *Not implemented*  *(Not in real-world database)* |
| *Any other diseases, metabolic dysfunction, physical examination finding, or clinical laboratory finding giving reasonable suspicion of a disease or condition that contraindicates the use of an investigational drug or that may affect the interpretation of the results or render the patient at high risk from treatment complications* | *Any other diseases, metabolic dysfunction, physical examination finding, or clinical laboratory finding giving reasonable suspicion of a disease or condition that contraindicates the use of an investigational drug or that may affect the interpretation of the results or render the patient at high risk from treatment complications* | *Any other diseases, metabolic dysfunction, physical examination finding, or clinical laboratory finding giving reasonable suspicion of a disease or condition that contraindicated the use of an investigational drug or that may have affected the interpretation of the results or rendered the patient at high risk from treatment complications* | *Not implemented*  *(Not in real-world database)* |
| *N/A* | *Patients with illnesses or conditions that interfere with their capacity to understand, follow and/or comply with study procedures* | *Illness or condition that may have interfered with a patient’s capacity to understand, follow, and/or comply with study procedures* | *Not implemented*  *(Not in real-world database)* |
|  | | | |
| ***Exclusion Criteria Related to Medications*** | | | |
| *NA* | *NA* | *Prior treatment with EGFR inhibitors or ALK inhibitors* | *Implemented* |
| *Any approved anti-cancer therapy, including chemotherapy, or hormonal therapy within 3 weeks prior to initiation of study treatment; the following exceptions are allowed:*   - ***Hormone-replacement therapy or oral contraceptives;*** - ***TKIs approved for treatment of NSCLC discontinued 7 days prior to randomization.***   *The baseline scan must be obtained after discontinuation of prior TKIs.* | *Any approved anti-cancer therapy, including hormonal therapy, within 21 days prior to initiation of study treatment; the following exceptions are allowed:*   - ***TKIs approved for treatment of NSCLC discontinued 7 days prior to randomization.***   *The baseline scan must be obtained after discontinuation of prior TKIs.* | *Any approved anti-cancer therapy, including hormonal therapy within 21 days prior*  *to initiation of study treatment.* | *Implemented*  *(Same)* |
| *Treatment with any other investigational agent or participation in another clinical study with therapeutic intent within 28 days prior to randomization* | *Treatment with any other investigational agent with therapeutic intent within 28 days prior to randomization* | *Treatment with any other investigational agent with therapeutic intent within 28 days prior to randomization* | *Not implemented*  *(Not necessary since patients receiving clinical study drug in a prior line will not be eligible.)* |
| *Prior treatment with CD137 agonists or immune checkpoint blockade therapies, anti-PD-1, and anti-PD-L1 therapeutic antibodies* | *Prior treatment with CD137 agonists or immune checkpoint blockade therapies, anti-PD-1, and anti-PD-L1 therapeutic antibodies* | *Prior treatment with CD137 agonists or immune checkpoint blockade therapies, antiPD-1, and antiPD-L1 therapeutic antibodies* | *Implemented*  *(Same)* |
| *Treatment with systemic immunostimulatory agents (including but not limited to IFNs, IL-2) within 4 weeks or five half-lives of the drug, whichever is longer, prior to randomization* | *Treatment with systemic immunostimulatory agents (including, but not limited to, IFNs, IL-2) within 4 weeks or five half-lives of the drug, whichever is longer, prior to randomization* | *Treatment with systemic immunostimulatory agents (including, but not limited to, interferons, interleukin 2) within 4 weeks or 5 half-lives of the drug, whichever was longer, prior to randomization* | *Not implemented*  *(Likely low prevalence)* |
| *Treatment with systemic immunosuppressive medications (including but not limited to* ***prednisone****, cyclophosphamide, azathioprine, methotrexate, thalidomide, and anti-TNF agents) within 2 weeks prior to randomization* | *Treatment with systemic immunosuppressive medications (including, but not limited to, corticosteroids, cyclophosphamide, azathioprine, methotrexate, thalidomide, and anti-TNF agents) within 2 weeks prior to randomization* | *Treatment with systemic immunosuppressive medications (including, but not limited to, corticosteroids, cyclophosphamide, azathioprine, methotrexate, thalidomide, and anti-TNF agents) within 2 weeks prior to randomization* | *Not implemented*  *(This criteria likely cannot be accurately implemented using data captured in the real-world database. Completeness of medications captured in structured orders is likely low, and describing exposure to those medications requires assumptions.)* |
|  | | | |
| ***Exclusion Criteria Related to Chemotherapy*** | | | |
| *Known history of severe allergic reactions to platinum-containing compounds or mannitol* | *Known history of severe allergic reactions to platinum-containing compounds or mannitol* | *History of allergic reactions to cisplatin, carboplatin, or other platinum-containing compounds* | *Not implemented*  *(Not in real-world database)* |
| *Known sensitivity to any component of nab-paclitaxel* | *Known sensitivity to any component of* ***paclitaxel*** *or nab-paclitaxel* | *N/A* | *Not implemented*  *(Not in real-world database)* |
| *N/A* | *N/A* | *Patients with hearing impairment (cisplatin)* | *Not implemented*  *(Not in real-world database)* |
| *Grade ≥ 2 peripheral neuropathy as defined by NCI CTCAE v4.0 criteria* | *Grade ≥ 2 peripheral neuropathy as defined by NCI CTCAE v4.0 (****paclitaxel and nab-paclitaxel****)* | *Grade 2 peripheral neuropathy as defined by NCI CTCAE v4.0 (****cisplatin****)* | *Not implemented*  *(Not in real-world database)* |
| *Known history of severe hypersensitivity reactions to products containing Cremophor® EL* | *Known history of severe hypersensitivity reactions to products containing Cremophor® EL* | *N/A* | *Not implemented*  *(Not in real-world database)* |

Notes: 1L = first-line therapy; ANC = Absolute Neutrophil Count; aPTT = activated partial thromboplastin time; ALT = alanine transaminase; ALK = anaplastic lymphoma kinase; AST = aspartate transaminase; CNS - central nervous system; CT = computerized tomography; CrCl = creatinine clearance; DNA = deoxyribonucleic acid; ECOG PS = Eastern Cooperative Group Performance Status; EGFR = epidermal growth factor receptor; HBcAb = hepatitis B core antibody; HBV = hepatitis B virus; HCV = hepatitis C virus; HBcAb = hepatitis B core antibody; HBsAg = hepatitis B surface antigen; HIV = human immunodeficiency virus; IFN = interferon; IL-2 = interleukin 2; INR = International normalized ratio; NA = not available; NSCLC = non-small cell lung cancer; OC = observational comparator; PCR = polymerase chain reaction; PD-1 = programmed cell death protein 1; PD-L1 = programmed death-ligand 1; RECIST = Response Evaluation Criteria in Solid Tumors; RNA = ribonucleic acid; TKI = tyrosine kinase inhibitor; TNF = tumor necrosis factor; Upper Limit of Normal = ULN.

**Supplementary Table 3.** Observational comparator attrition table.

| **Criteria** | **Number of patients** |
| --- | --- |
| Total patients in the cohort (Dec 2020) | 66520 |
| Line start date is between ‘2015-04-16’ and ‘2017-05-31’ | 15009 |
| 1L after advanced diagnosis | 12310 |
| Patients with multiple primary tumors excluded | 12190 |
| Age >= 18 years-old | 12190 |
| ECOG PS 0, 1, or missing at baseline ^a^ | 10532 |
| Histology known | 10052 |
| With structured activities within 90 days of advanced diagnosis | 9255 |
| Without abnormal labs or missing within a -30 to +7 window around 1L start date | 8412 |
| Without EGFR mutation or missing at any time up to 7 days after 1L start | 7493 |
| Without ALK mut or missing at any time up to 7 days after 1L start | 7313 |
| No exposure to chemotherapy within prior six months | 7004 |
| No exposure to prior CIT | 6984 |
| With the regimens of interest in 1L | 3340 |

Notes: 1L = first-line therapy; ALK = anaplastic lymphoma kinase; CIT = cancer immunotherapy; ECOG PS = Eastern Cooperative Group Performance Status; EGFR = epidermal growth factor receptor;

^a^ The closest ECOG value within a -30 to +7 window around 1L start date was used, taking the highest if multiple observations occurred on the same day

**Supplementary Table 4.** Baseline characteristics by trial.

| **Variable** | **Categories** | **RCT** | | | | **OC N=3340** |
| --- | --- | --- | --- | --- | --- | --- |
|  |  | **Total**  **N=849** | **IMpower 130**  **N=228** | **IMpower 131**  **N=340** | **IMpower 132**  **N=281** |  |
| Age group (years), n(%) | < 65 | 435 (51.2) | 114 (50.0) | 156 (45.9) | 164 (58.4) | 1222 (36.6) |
|  | ≥ 65 and < 75 | 322 (37.9) | 83 (36.4) | 145 (42.6) | 94 (33.5) | 1268 (38.0) |
|  | ≥ 75 | 92 (10.8) | 30 (13.2) | 39 (11.5) | 23 (8.2) | 850 (25.4) |
| Gender, n(%) | Female | 248 (29.2) | 94 (41.2) | 63 (18.5) | 91 (32.4) | 1457 (43.6) |
| Race, n(%) | Asian | 105 (12.4) | 3 (1.3) | 37 (10.9) | 65 (23.1) | 46 (1.4) |
|  | White | 699 (82.3) | 210 (92.1) | 290 (85.3) | 199 (70.8) | 2373 (71.0) |
|  | Other | 45 (5.3) | 15 (6.6) | 13 (3.8) | 17 (6.0) | 921 (27.6) |
| ECOG PS, n(%) | 0 | 314 (37.0) | 91 (39.9) | 110 (32.4) | 113 (40.2) | 714 (21.4) |
|  | 1 | 532 (62.7) | 136 (59.6) | 229 (67.4) | 167 (59.4) | 1179 (35.3) |
|  | 2 | 1 (0.0) | 1 (0.4) | 0 (0.0) | 0 (0.0) | 0 (0.0) |
|  | Unknown | 2 (0.2) | 0 (0.0) | 1 (0.3) | 1 (0.4) | 1447 (43.3) |
| Metastatic diagnosis, n(%) | De novo Stage IV | 706 (83.2) | 188 (82.5) | 270 (79.4) | 248 (88.3) | 2118 (63.4) |
|  | Recurrent disease | 143 (16.8) | 40 (17.5) | 70 (20.6) | 33 (11.7) | 1221 (36.6) |
| Smoking history, n(%) | No | 69 (8.1) | 17 (7.5) | 24 (7.1) | 28 (10.0) | 257 (7.7) |
|  | Yes | 780 (91.9) | 211 (92.5) | 316 (92.9) | 253 (90.0) | 3070 (91.9) |
|  | Unknown | 0 (0.0) | 0 (0.0) | 0 (0.0) | 0 (0.0) | 13 (0.4) |
| Histology, n(%) | Non-squamous | 509 (60.0) | 228 (100.0) | 0 (0.0) | 281 (100.0) | 2278 (68.2) |
|  | Squamous | 340 (40.0) | 0 (0.0) | 340 (100.0) | 0 (0.0) | 1062 (31.8) |
| Time from initial diagnosis to index date (months), (median [IQR]) |  | 1.41 [0.92, 2.89] | 1.58 [1.08, 3.08] | 1.41 [0.91, 3.96] | 1.35 [0.92, 2.43] | 1.25 [0.79, 2.27] |
| Treatment, n(%) | Carboplatin+Pacli/Nab-pacli | 568 (66.9) | 228 (100.0) | 340 (100.0) | 0 (0.0) | 1877 (56.2) |
|  | Platinum+Pemetrexed | 281 (33.1) | 0 (0.0) | 0 (0.0) | 281 (100.0) | 1463 (43.8) |

Notes: ECOG PS = Eastern Cooperative Group Performance Status; OC = observational comparator; RCT = randomized clinical trial.

**Supplementary Table 5.** Average* distribution of IPCW stabilized weights by group.

|  | **RCT** | **OC** |
| --- | --- | --- |
| Mean | 0.94 | 0.99 |
| SD | 0.84 | 0.5 |
| Median | 0.94 | 0.9 |
| Minimum | 0.28 | 0.39 |
| Maximum | 4.22 | 5.66 |

Notes: IPCW = inverse probability of censoring weighting

*The requirement of a mean of one applies to the estimated weights at each time point, but, as a simplification, we pooled the estimated weights from all time points in the study.
